# Impact of Extreme Weather Disasters on China’s Barley Industry under the Background of Trade Friction—Based on the Partial Equilibrium Model

**DOI:** 10.3390/foods11111570

**Published:** 2022-05-26

**Authors:** Jingyi Liu, Xiande Li

**Affiliations:** Institute of Agricultural Economics and Development, Chinese Academy of Agricultural Sciences, Beijing 100081, China; lwt2020215@163.com

**Keywords:** trade friction, extreme weather disasters, barley trade, partial equilibrium

## Abstract

The world has entered a compound risk era with multiple crises, and the adverse impact of trade friction and extreme weather disasters on China’s barley import has become increasingly prominent. In this context, this study uses superimposed epoch analysis and partial equilibrium model to evaluate the impact of extreme weather disasters in China’s major barley-exporting countries on China’s barley industry in the course of China–Australia trade friction. The results show that: (1) extreme weather disaster caused barley production in France and Canada to decrease by 7.95% and 18.36% respectively; (2) when the two external shocks occur at the same time, China’s barley import volume tends to decline compared with the basic scenario, the import price rises sharply, there are certain trade-diverting effects in barley import, and China’s imports from countries not affected by extreme weather disasters will increase to a certain extent; (3) China’s barley production remains at a low rate of growth and is vulnerable to external shocks, facing certain import risks. This study provides important policy implications for preventing import risks and ensuring the sufficient supply of domestic barley.

## 1. Introduction

Due to local conflicts, economic shocks, and extreme weather disasters, 155 million people worldwide have fallen into food insecurity, threatening to trigger a global food crisis [1]. Extreme weather disasters exacerbate the vulnerability of the global food supply system with their uncertainty [2,3], resulting in the stagnation or decline of some global food production [4], which has become one of the biggest threats to food security in the 21st century [5,6]. Barley is one of the most important cereals in feed and malt production. Compared with other crops, barley is highly tolerant to different and extreme environmental conditions [7,8], and is considered a food-secure crop [9]. However, in recent years, climate warming and frequent extreme weather disasters have directly jeopardized the stability of barley production, and even caused a serious reduction in barley production in some years [10]. For example, extreme weather caused a 26% decrease in barley production in France [11]. From 1980 to 1995, extreme droughts became the culprit for the 4.8% decline in barley production in western Kazakhstan [12]. In 2007, the extreme dry weather caused a 62.23% reduction in barley production in Australia, which seriously affected the global barley supply market. In the next decade, climate change is expected to be intensified [13]. It is predicted that the global drought index will rise by 52.15% by 2050, and the number of drought-affected areas will increase by 28.6% by 2100 [14]. The impact of extreme weather on the main barley-producing areas in the world are different. In this regard, many scholars have predicted barley production in view of possible climate changes in the future. For example, a 4-degree Celsius increase in global temperature will be disastrous to the barley production in Finland, with up to a 50% reduction in production [15]. Some scholars believe that the rise of temperature can bring potential for the increase of crop yield, which is conducive to the increase of yield per unit area of crops such as soybean and corn, but can weaken the competitive advantage of barley, which indirectly leads to the reduction of barley harvested area [16]. Research data show that droughts are likely to affect barley production in England, China, Australia, and other countries to varying degrees in the future [17,18,19]. It is predicted that changes in rainfall in Ireland will reduce barley production by 4.5 tons per hectare in 2055 [20]. The reduction in barley production will eventually affect the global industrial chains of feed and beer [21,22]. The impact of extreme weather in production is bound to trigger market fluctuations and soaring prices [23], prompting international trade to redistribute global production and consumption [24].

China is the world’s largest importer and consumer of barley. In 2015, China signed a free trade agreement with Australia, imposing a zero tariff on imported barley originating from Australia. Since 2015, Australian barley has begun to flow into the Chinese market, and Australia has become China’s largest barley exporter [25]. Australia’s barley exports to China pose a threat to China’s self-sufficiency and import diversification policies [26]. Driven by ensuring food security, on 19 November 2018, the Chinese Ministry of Commerce decided to file an anti-dumping investigation on imported barley originating from Australia and finally concluded that the imported Australian barley hurt the domestic barley industry in China. As a result, a countervailing duty of 6.9% and an anti-dumping duty of 73.6% were imposed on importing Australian barley for a period of five years from 19 May 2020. After the outbreak of this trade friction, China’s barley import was further concentrated in Canada and France.

Though grain trade is an effective way to ensure the global grain supply [27], it also increases the risk of external interference [28]. When grain trade is dominated by only a few countries, it will greatly amplify the vulnerability of the national food system towards external shocks such as global weather disasters and political instability, and thereby, affect the national food security [29]. Therefore, how will China’s barley import change? Will China still be able to obtain sufficient barley supply in the international market? In order to answer the above questions, we first apply a superposition epoch analysis to evaluate the impact of extreme weather disasters on barley production in China’s major exporting countries from 1961 to 2020; second, we construct a multi-country partial equilibrium model to simulate the impact of external shocks on China’s barley import structure and domestic barley production. Existing studies mainly focus on predicting the impact of a single extreme event on barley production, and to our knowledge, thus far, there have been no analyses of multiple risks’ impact on barley production and trade. This study exposes the multiple risks faced by China’s barley import trade to ensure a stable and sufficient supply of barley, through the simulation of different scenarios.

## 2. Import Structure and Disaster Occurrence

### 2.1. Descriptive Analysis of China’s Barley Import Pattern

**Figure 1:**
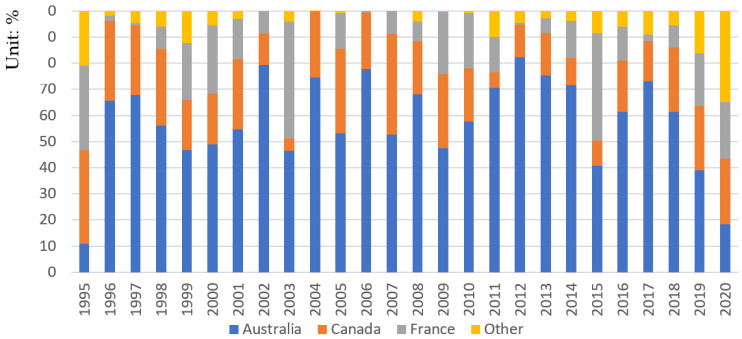
Changes in import proportion of major exporting countries of barley in China from 1995 to 2020 (unit: %).

Figure 1 shows the structural characteristics of China’s barley import from 1995 to 2020. From 1995 to 2020, China’s barley import trade was monopolized by Australia, Canada, and France. Before the trade friction between China and Australia, China’s barley imports from Australia, Canada, and France accounted for more than 95% of the total imports, and even reached 100% from 2004 to 2007. From 2018 to 2020, the trade friction between China and Australia led to a sharp decline in China’s barley imports from Australia. However, by 2020, the proportion of barley imports from Australia, Canada, and France still accounted for 65.21% of the total import.

In 1995, China’s barley imports from Australia accounted for only 10.88%. From 1996 to 2018, China’s imports from Australia began to increase significantly, accounting for an average of 63.14% of the total imports. It reached a historical peak of 82.22% in 2012. In 2015, the China–Australia free trade agreement was officially signed, and China implemented a zero-tariff policy on importing Australian barley. Since then, the quantity of barley imported by China from Australia has maintained a high level. However, the extremely low price of Australian barley has impacted China’s domestic barley industry, resulting in a precipitous decline in China’s barley harvested area. Therefore, in November 2018, the Chinese Ministry of Commerce decided to conduct an anti-dumping investigation on imported barley originating in Australia. Since the anti-dumping investigation began, Chinese beer manufacturers began to adjust their import sources and reduce their imports from Australia. By 2019, China’s barley imports from Australia fell to 39.09%. In May 2020, the Chinese Ministry of Commerce believed that the import of barley from Australia had caused substantial damage to the domestic barley industry. Therefore, it was ruled to impose 73.60% anti-dumping and 6.90% anti-subsidy tariffs on importing Australian barley, causing China’s barley imports from Australia to decrease significantly.

Canada is famous for producing beer barley [30], and it is the second-largest barley exporter to China. From 1995 to 2002, China’s barley imports from Canada were relatively stable, accounting for an average of 24.67% of the total imports. In 2003, China increased its barley imports from France. As a result, the proportion of imports from Canada decreased to 4.89%. From 2004 to 2010, China’s barley imports from Canada began to increase, accounting for an average of 26.70% of the total imports. From 2011 to 2017, with the substantial increase of China’s barley imports from Australia, the proportion of imports from Canada showed a downward trend, accounting for an average of 12.05% of the total imports. The outbreak of trade friction between China and Australia then had a trade-diverting effect on China’s barley imports. Since then, Canada becomes the largest barley exporter to China, with the proportion of barley imports from Canada increasing to 24.95%.

Compared with Australia and Canada, the quantity of barley imported by China from France is relatively small and unstable. The main reason is that French barley has a lower leaching rate, which is not suitable for Chinese beer production. However, since the outbreak of trade friction between China and Australia, China’s barley imports from France have increased significantly. In 2020, the amount of barley imported from France accounted for 21.77% of the total imports.

It can be seen from the above analysis that after the trade friction between China and Australia, China’s barley imports are more concentrated in France and Canada. The stability of barley production in the above two countries is very important to the security of China’s barley imports. Therefore, this paper focuses on the impact of extreme weather disasters on barley production in Canada and France.

### 2.2. Natural Disasters in France and Canada

According to EM-DAT statistics, during the barley planting season, there were 28 and 59 natural disasters that occurred in the main barley-producing areas in France and Canada respectively from 1961 to 2020 (Table 1). In France, floods occurred the most, followed by droughts. The above two extreme weather disasters accounted for 42.68% and 25.00% of the total natural disasters, respectively. In Canada, storm and floods occurred the most, accounting for 33.89% and 28.81% of total natural disasters, respectively. In general, the most frequent natural disaster in France and Canada are floods, storm, droughts, and extreme weather. The sum of the above four natural disaster accounts for 71.67%, 51.75%, 31.78%, and 22.76% of the total number of natural disasters, respectively. According to relevant studies, barley has relatively low requirements for soil quality [31], but when the soil moisture is too high or too low, it will have a negative impact on barley yield [32,33]. Especially in the two golden periods of early and late heading, droughts or floods have the most serious impact on barley yield [34]. At the same time, extremely low or high humidity will also increase the probability of barley infection with pathogens [35], thus affecting the yield. Extreme weather disasters, including floods and droughts, affect crop yield in mid-latitude countries such as France and Canada the most [36,37,38]. Therefore, this study selects two extreme weather disasters (i.e., floods and droughts) to analyze the reduction of barley yield in the main barley-producing areas of France and Canada during the barley planting season.

## 3. Materials and Methods

### 3.1. Method

#### 3.1.1. Superimposed Epoch Analysis

This paper uses superimposed epoch analysis (SEA) to assess the impact of two extreme weather events, flood and drought, on barley production in France and Canada, the major barley-exporting countries for China. SEA is a statistical method that is commonly used to enhance the signal in time series data to analyze the impact of a particular event. At the same time, it is able to eliminate noise due to extraneous variables [39]. SEA suppresses noise by averaging the impact of events, which effectively amplifies the relative magnitude of the response signal and extracts the impact of a specific event from the accompanying noise [40]. The assessment using SEA begins by creating a composite matrix, which is a fixed window of continuous observations plotted over a continuous time series before, during, and after the “event”. Then, the average of the composite matrix is used as the “epoch response”. Finally, the statistical significance of the “response” is determined using a randomization scheme, which assesses the likelihood of the “response” occurring by comparing it with the null hypothesis [41]. SEA has been used in a wide range of applications in space and environmental sciences. For example, it is used in studies of climatology [42,43,44], or to determine the correlation between fire events and soil moisture conditions [45,46,47,48,49,50].

#### 3.1.2. Partial Equilibrium Model

##### Settings

Based on the partial equilibrium theory, this paper draws lessons from the China agricultural industry model (CASM) developed by the Institute of Agricultural Economics and Development of the Chinese Academy of Agricultural Sciences [51]. On the one hand, CASM can predict the supply, demand, price, and import and export trade of agricultural products in the future [52]. On the other hand, it can be widely used to simulate and analyze the impact of policy changes on China’s agricultural product market. This study divides the trade sector in the CASM into Australia, France, Canada, and other countries (barley-exporting countries other than Australia, France, and Canada), so as to calculate the import of Chinese Barley from major countries.

1. Production Equations. The yield of crops is determined by the yield per unit area and harvested area. The yield per unit area of barley is related to the productive factor and the level of growing techniques. Growing techniques are generally stable in the short term. The harvested area of barley is mainly determined by the comparative effectiveness of barley and other crops. If growing barley can bring more benefits, the harvested area of barley will increase.
  (1) $$QCHN_{t}=A_{t}\times Y_{t}$$
  (2) $$LnA_{t}=\alpha^{A}+e_{DBP}^{A}LnDBP_{t}+e_{DCP}^{A}LnDCP_{t}+\sum e_{DOCP}^{A}LnDOCP_{t}$$
  (3) $$LnY_{t}=\alpha^{Y}+e_{DBP}^{Y}LnDBP_{t}$$
  where *QCHN* is the yield of barley, *A* is the barley harvested area, *Y* is the yield per unit area of barley, *DBP*, *DCP*, and *DOCP* are the price of barley, corn, and other agricultural products (i.e., rice, wheat, potato, soybean, rapeseed, peanut, cotton, sugarcane, sugar, beet, apple, other fruits and vegetables), respectively, α is the intercept, *e* is the elasticity, and *t* is the time variable.
2. Demand Equations. Barley is mainly demanded for feed and processing. Both of these demands are influenced by barley prices and domestic income levels. The equation for the demands of barley is as follows:
  (4) $$LnDBP_{t}=\alpha^{DBP}+\beta_{PGDP}^{DBP}Ln(PGDP_{t}/CPI_{t})+\beta_{DDCN}^{DBP}LnDDCN_{t}$$
  where *DBP* is the demand, *PGDP* and *CPI* reflects the level of per capital income, and *DDCN* is China’s barley demand.
3. Trade Equations. This study uses the barley yield of exporting countries to represent their export capacity, and uses China’s barley import price and domestic price to reflect China’s import demand. It is considered that there is uncertainty in international trade, and the significant increase or decrease of China’s barley import in individual years will affect the parameter estimation, so the influence can be eliminated by setting dummy variables. At the same time, China’s barley import countries are divided into Australia, France, Canada, and other countries. The equations for the China’s barley import are as follows:
  (5) $$LnIM_{it}=\alpha^{IM_{i}}+\beta_{Q}^{{}^{IM_{i}}}LnQ_{it}(1-Z_{i})+\beta_{DBP}^{{}^{IM_{i}}}LnDBP_{t}+\beta_{IBP}^{{}^{IM_{i}}}LnIBP_{it}+\beta_{DZ}^{{}^{IM_{i}}}DZ_{it}+\beta_{DJ}^{{}^{IM_{i}}}DJ_{it}$$
  where *IM* represents China’s barley imports, *i* represent Australia, Canada, France, and other countries, *Q* represents the barley production, *IBP* represents the import price of barley in China, *DZ* and *DJ* represent the dummy variables setting to eliminate the positive and negative shocks in each country, respectively, and *Z* represents percentage of impact of extreme weather disasters on barley yield.
4. Price Linkage Equations. There is a correlation between China’s barley import price and the domestic price. China’s barley import price is mainly affected by the exchange rate and corn price. The equations can be given as:
  (6) $$LnIBP_{it}=\alpha^{IBP_{i}}+\beta_{DBP}^{IBP_{i}}LnDBP_{t}+\beta_{EX}^{IBP_{i}}LnEX_{it}+\beta_{DCP}^{IBP_{i}}LnDCP_{t}$$
  where *EX* represents the exchange rates of Chinese Yuan (RMB) with Australian Dollar (AUD), Canadian Dollar (CAD), Euro (EURO), and US Dollar (USD) in period *t*, respectively.
5. Market Clearing. Since China has been in a state of net import for a long time, it is assumed that the market will reach the clearing state when the sum of domestic output and import is equal to domestic consumption. This can be given as:
  (7) $$DDCN_{t}=QCHN_{t}+\sum IM_{it}$$

##### Parameter Estimation

In this paper, relevant parameters of the production module in the Chinese barley international trade model are set based on the China agricultural sector model (CASM) by Chinese Academy of Agricultural Sciences.

The parameters of demand equations, trade equations, and price linkage equations in the China’s barley import trade model are obtained by regression. The estimation results show that the fitting degree (*R*^2^) of most equations is high, indicating a high overall fitting degree. Moreover, most of the variables in the model are significant and consistent with the economic principles, indicating that the independent variables have satisfactory explanatory power (see Supplementary Material).

### 3.2. Data

Table 2 and Table 3 are the data sources and descriptive analysis of variables. The production data of China’s barley harvested area, yield per unit area, and the yield of Canada and France are obtained from FAO Database [53]. China’s barley import quantity and import price are obtained from UN Comtrade database [54], in which the import price is the ratio of total import volume to import quantity. We used data over the period 1995–2020. In order to eliminate the influence of abnormal fluctuation on parameter estimation to the greatest extent, the average import quantities of the current year and the previous and subsequent years are used to replace the import quantity of the current year. The data of extreme weather disasters in Canada and France between 1961 and 2020 are obtained from the EM-DAT database [55].

### 3.3. Scenario Settings

In France, floods and droughts caused a 7.7% and 8.19% reduction in barley production, respectively, as calculated by the superimposed epoch analysis method. The average effect of droughts and floods on barley yield was 7.95%. Since the two extreme weather disaster have similar effects on barley yield, the average value of 7.95% is taken as the impact of extreme weather disasters on barley yield in France. Similarly, droughts and floods reduced Canada’s barley production by 16.24% and 20.53%, respectively, with an average of 18.36%, which is regarded as the impact of extreme weather disaster (floods or droughts) on Canada’s barley production. Based on the above analysis, the basic scenario is set as follows: China imposes a “zero tariff” policy on barley imported from Australia and a 3% tariff on barley imported from France, Canada, and other countries (Table 4). Scenario 1 is set as follows: China imposes 80.50% anti-dumping and countervailing duties on barley imported from Australia, and extreme weather disasters reduce the production of barley in France by 7.95%; Scenario 2 is set as follows: China imposes 80.50% anti-dumping and countervailing duties on barley imported from Australia, and extreme weather disasters reduce Canada’s barley production by 18.36%.

## 4. Results and Discussion

### 4.1. The Impact of Drought and Floods on Barley Production in France and Canada

This study uses the superposition epoch analysis to calculate the yield reduction caused by floods and droughts in the main barley-producing areas and barley-planting seasons in France and Canada from 1961 to 2020. In France, two extremely high temperatures occurred in 1990 and 2003, which had negative impacts on barley production. In order to comprehensively measure the loss of barley yield caused by the extreme weather disasters, extremely high temperatures are classified as droughts.

According to the statistics of EM-DAT, during the barley-planting season from 1961 to 2020, there were twelve floods and seven droughts occurred in the main barley-producing areas of France, of which four floods and droughts caused a serious reduction in barley production. Specifically, the average impact of floods on barley yield, area, and yield in France were −8.39%, 1.04%, and −7.70%, respectively (Table 5). The average effects of droughts on barley yield, area, and yield in France were −8.18%, −0.27%, and −8.19%, respectively. Through a comparison, it can be seen that the two extreme weather disaster of floods and droughts have similar effects on yield per unit area and yield of barley. However, the impact of floods and droughts on yield is less than that on yield per unit area. At the same time, floods and droughts have a positive impact on barley-harvested area in some years. The main reason is that the artificial expansion of barley-harvested area could compensate the impact of weather disasters on barley yield to a certain extent. For example, the floods in 2001 reduced the yield of barley by 11.70%, but the barley harvested area in France increased by 7.38% year-on-year in 2001. Thus, the floods only reduced barley production by 5.27%.

According to the statistics of EM-DAT, from 1961 to 2020, there were seventeen floods and four droughts that occurred in the main barley-producing areas of Canada during the barley-planting season, of which four floods and three droughts resulted in a significant reduction in local barley production (Table 6). Specifically, the average effects of droughts on barley yield per unit area, harvested area, and yield were −16.96%, −3.52%, and −20.53%, respectively. As high latitudes and the Arctic are more sensitive to climate warming, Canada is warming twice as much as the global average, and extreme weather disasters will devastate crop production in Canada [56]. For example, in 1961, although the barley acreage increased, the droughts caused a 35.07% decrease in barley yield per unit area, which eventually led to a 32.09% reduction in barley production. Canada is located in the Atlantic Ocean and is extremely vulnerable to flooding [57]. Although floods occurred more frequently in Canada, they have less impact on barley production than droughts. The main reason is that 90% of barley-growing areas are located in southern Canada, which is the main grain-producing area in Canada. The soil in this area is fertile, but the climate is dry and there is less rainfall. Therefore, droughts have a more serious impact on the barley yield in Canada [58].

### 4.2. Estimation Results

China imposes 80.50% anti-dumping and countervailing duties on imported barley from Australia. The yield of barley in France decreases by 7.95% due to floods or droughts. Compared with the basic scenario, when the above two external shocks occur at the same time, China’s barley import volume will decrease by 9.65%, and the weighted average barley import price will increase by 12.06% (Table 7). Due to the severe decline of barley production in France caused by extreme weather disasters, China’s barley imports from France decreased by 17.30%. As China imposed high tariffs on imported barley originating in Australia, China’s barley imports from Australia decreased significantly by 41.68%. Under the influence of the trade-diversion effect, China’s barley imports from Canada and other countries increased by 4.32% and 2.5%, respectively. The decrease of China’s barley imports from France and Australia is bound to increase the China’s barley import price. However, it is estimated that in addition to the sharp rise in import price from Australia, the increase from France, Canada, and other countries is small. Compared with the basic scenario, China’s import prices from Australia, Canada, France, and other countries increased by 84.52%, 1.18%, 1.43%, and 1.90%, respectively.

Compared with the basic scenario, when the two external shocks occur at the same time, it will increase domestic production by 1.24% and the domestic price by 3.78% (Table 8). With the decrease of total supply, the barley consumption decreased by 8.6%.

China imposes 80.50% anti-dumping and countervailing duties on imported barley from Australia. The yield of barley in Canada decreases by 18.36% due to floods or droughts. Compared with the basic scenario, when the above two external shocks occur at the same time, China’s barley imports decrease by 9.15% and the weighted average import price increases by 11.28% (Table 9). Extreme weather disasters damaged Canada’s barley production, reducing China’s barley imports from Canada by 10.05%. Trade frictions between China and Australia led to a sharp drop of 41.66% in China’s barley imports from Australia. Compared with Scenario 1, Scenario 2 has a lower trade diversion effect. China’s barley imports from France and other countries increased only by 2.16% and 2.60%. China imposed high import tariffs on Australia, which led to a sharp rise in import price, reaching 84.67%. Compared with the barley import price in Australia, import prices from Canada, France, and other countries slightly increased by 1.22%, 1.49%, and 1.97%, respectively.

Compared with the basic scenario, when the two external shocks occur at the same time, it will increase domestic production by 1.28% and the domestic price by 3.81% (Table 10). With the decrease of total barley supply, the domestic barley consumption decreased by 8.14%.

### 4.3. Comparison of the Simulated Results with the Actual Situation

According to the China Customs Database, in November 2021, China’s barley imports from Australia, Canada, and France were priced at 248.56 USD/ton, 234.98 USD/ton, and 241.01 USD/ton, respectively. When China imposes an 80.5% tariff on barley imports from Australia, the import price of barley from Australia will be about 448.65 USD/ton. Compared with the import prices of barley from France and Canada in the same period, Australia lost its price advantage. In addition, barley, as an important raw material for feed, has a close substitution relationship with sorghum and corn. Import prices of sorghum and corn in November 2021 were 331.56 USD/ton and 308.86 USD/ton, respectively. The import price of barley in Australia is much higher than that of sorghum and corn in the meantime. Therefore, Chinese enterprises temporarily suspended the import of barley from Australia in December 2021.

The trends in the simulated results of the two scenarios set up in the study are generally consistent with the actual situation. In the simulated scenario, the high tariff, which increases the price of China’s barley imports from Australia significantly, brings about a sharp decrease in China’s imports from Australia. In the real situation, firms would abandon imported barley from Australia and be in favor of other import sources in order to maximize their profits.

After calculation, the increase of domestic barley yield under the simulation scenario is very small. The simulation results are highly consistent with the change trend of domestic barley yield. This shows that even if there is an external shock, the increase of domestic barley production is limited. The main reason is that the barley industry has no competitive advantage in China. As a kind of coarse grain, barley has not been supported by agricultural policies in China. The market price of domestic barley is about 7.5% lower than that of wheat. Therefore, the benefits of growing barley are less. Farmers gradually give up the cultivation of barley and plant wheat instead. At the same time, before China imposed anti-dumping and countervailing tariffs on Australia, China’s import price from Australia was lower than the domestic market price. In order to reduce production costs, domestic enterprises tend to use imported barley. Therefore, although barley has strong adaptability to the environment, the barley industry does not have competitive advantages in China, so it is difficult to improve the domestic barley production in the short term.

## 5. Conclusions and Policy Implications

Based on the data of extreme weather disasters in Canada and France from 1961 to 2020, as well as China’s barley import trade data and domestic production data in 2020, this study analyzes the impact on China’s barley import and domestic production caused by extreme weather disasters in major barley-exporting countries under the background of trade friction between China and Australia. The results show that when China both imposes an 80.5% anti-dumping and countervailing policy on Australian barley, and extreme weather disasters dampen Canada’s barley production by 18.36%, China’s total barley imports will decrease by 9.35% and the weighted average import price will rise by 11.86% compared with the base scenario. Among them, China’s barley imports from Australia and Canada fell by 41.66% and 10.05%, respectively. Under the trade-diversion effect, China’s barley imports from France and other countries increased by 2.16% and 2.60%, respectively, and China’s import prices from Australia, Canada, France, and other countries increased by 84.67%, 1.22%, 1.49%, and 1.97%, respectively. Moreover, the domestic barley production and price increased by 1.28% and 3.81% respectively, and the consumption decreased by 8.31%. When China imposes an 80.5% anti-dumping and countervailing policy on Australian barley, and the production of barley in France is reduced by 7.95% due to extreme weather disasters, compared with the basic scenario, China’s total barley import volume will be reduced by 9.77% and the weighted average import price will rise by 12.10%. Among them, China’s barley imports from Australia and France decreased by 41.68% and 17.30%, respectively. Due to the trade-diversion effect, China’s barley imports from Canada and other countries increased by 4.32% and 2.50%, respectively. China’s import prices from Australia, Canada, France, and other countries rose by 84.54%, 1.18%, 1.43%, and 1.97%, respectively. Meanwhile, the domestic production and price increased by 1.24% and 3.78%, respectively, and the consumption decreased by 8.69%.

Unlike previous studies on the impact of extreme natural disasters on barley production and trade, the simulation results of this study include the dual impacts of China–Australia trade frictions and extreme weather disasters on China’s barley import, which can reflect the potential risks faced by China’s barley import in a more comprehensive manner. It provides a reference for China to establish and improve the barley production and trade-support policy system, which is of great practical significance to better prevent the adverse effects of external shocks and ensure the adequate supply of China’s barley market.

It is noteworthy that when Chinese barley imports suffered from a major external shock, it did not stimulate the increase of domestic barley production. It indicates that the limited domestic resource endowment makes it difficult to increase barley production significantly in the short term, and the international barley market is still the main source to supply domestic barley demand. It also exposes the weakness of China’s domestic barley industry in coping with trade risks. Therefore, we have the following policy recommendations: First, on the basis of stabilizing existing import sources, actively expand new barley trade partners, and especially strengthen trade with countries with large barley-production potential. Second, establish an early warning system of barley prices and production costs at home and abroad, improve the monitoring, and strengthen the prediction and analysis of the global barley production, marketing situation, and international market prices to guide domestic enterprises to reasonably arrange production and prepare for external shocks in advance. Third, establish business alliances to improve the bargaining power of international trade, especially with major barley importers such as Japan, Saudi Arabia, and the Netherlands to give full play to the negotiating advantages of “big buyers” in barley international trade and achieve win-win results. Fourth, establish a compound new agricultural management system according to local conditions and encourage small farmers to actively participate in modern agricultural production. This can be done by reducing the production cost and transaction cost of barley, so as to improve the yield of barley and the income of farmers, and the same time, develop contract farming to reduce the impact of market uncertainty risk on barley production and stabilize barley supply.

In view of the limitations of this study, future research can further extend the following two aspects. Firstly, this study only simulates and compares the impact of “no external shocks” and “two external shocks at the same time” on China’s barley import trade and domestic production. More studies are warranted in forecasting the impact of external shocks on China’s barley import trade and production in the next few years, or even decades. Secondly, this study only selects two external shocks—trade frictions and extreme weather disasters—and only Australia, France, and Canada are selected as major exporting countries. Since Ukraine and Russia are China’s emerging partners on barley trade, research on the impact of the outbreak of the Ukraine-Russia conflict on China’s barley imports is also needed.

## Figures and Tables

**Table 1:** Overview of natural disasters in main producing areas of major barley-exporting countries (1961–2020).

	France		Canada	
	Frequency	Proportion	Frequency	Proportion
Flood	12	42.86	17	28.81
Storm	5	17.86	20	33.89
Drought	7	25.00	4	6.78
Extreme Temperature	4	14.29	5	8.47
Wild Fire	0	0	10	16.95
Landslide	0	0	1	1.69
Volcanic Activity	0	0	0	0
Pest	0	0	0	0
Infectious Diseases	0	0	2	3.39
Earthquake	0	0	0	0
Total Disaster	28	/	59	/

**Table 2:** Variables and data source.

Variable		Unit	Symbol	Data Sources
Endogenous variable	Barley production in China	10^4^ Ton	QCHN	FAOSTAT
	Barley planting area in China	10^4^ hectare	A	FAOSTAT
	Barley yield per unit area in China	Ton per hectare	Y	FAOSTAT
	barley market price in China	USD/Ton	DBP	FAOSTAT
	Barley consumption in China	10^4^ Ton	DDCN	FAOSTAT
	Chinese barley imports	10^4^ Ton	IM_i_	Un Comtrade
	Import barley price	USD/Ton	IBP_i_	Un Comtrade
Exogenous variable	Market prices of other agricultural products in China	USD/Ton	DOPC_i_	CASM
	China’s per capita GDP	USD	PGDP	NBS
	China Consumer Price Index	-	CPI	NBS
	Barley production in other countries	10^4^ Ton	Q_i_	FAOSTAT
	Positive external impact	-	DZ	-
	Negative external impact	-	DJ	-
	Exchange rate	-	EX_i_	World bank
	corn market price in China	USD/Ton	DCP	FAOSTAT
	Effect of extreme weather disasters on yield reduction rate of barley	%	Z	-

**Table 3:** Variables’ descriptive statistics.

Variable	Mean	Standard Deviation	Minimum	Maximum
DBP	217.02	86.45	95.41	330.94
DDCN	526.71	125.01	327.41	777.10
PGDP	12,537.51	9665.03	2362.30	32,189.00
CPI	102.77	3.58	98.60	117.10
IMa	138.40	80.24	64.97	324.07
IMc	57.31	39.80	25.94	175.48
IMf	49.26	54.33	4.17	190.45
IMo	31.50	29.94	7.32	133.76
Qa	783.93	209.56	386.48	1350.60
Qc	1039.46	237.45	711.68	1556.20
Qf	1075.58	140.77	759.03	1356.54
Qo	11,179.73	839.73	9535.19	12,510.68
IBPa	230.39	68.69	134.37	455.23
IBPc	247.95	77.59	148.30	439.55
IBPf	225.73	77.59	113.94	452.39
IBPo	239.75	77.20	148.73	439.80
EXa	5.48	0.68	4.29	6.66
EXc	5.85	0.68	4.87	7.11
EXf	8.64	1.09	6.94	10.42
EXo	7.37	0.86	6.14	8.35
DCP	218.65	93.81	115.94	389.09

**Table 4:** Design of the extreme natural disaster simulation scheme.

Programs	Country	Impact on Yield	Tariff
Basic scenario	-	-	Zero tariff on barley imported from Australia; 3% tariff on barley imported from other countries
Scenario 1	France	−7.95	80.5% tariff on barley imported from Australia; 3% tariff on barley imported from other countries
Scenario 2	Canada	−18.36	80.5% tariff on barley imported from Australia; 3% tariff on barley imported from other countries

**Table 5:** Effects of droughts and floods on yield per unit area, harvested area, and yield of barley in France (unit: %).

	Disaster Year	Impact on Yield per Unit Area	Average Impact	Impact on Harvested Area	Average Impact	Impact on Yield	Average Impact
Drought	1989–1990	−1.55	−8.18	−0.18	−0.27	−0.16	−8.19
	2003	−16.76		7.51		−10.50	
	2018	−6.23		−8.14		−13.90	
Flood	1993–1994	−1.67	−8.39	−2.83	1.04	−4.65	−7.70
	2001	−11.70		7.38		−5.27	
	2005	−1.01		−2.83		−3.76	
	2016	−19.16		2.45		−17.10	

**Table 6:** Effects of droughts and floods on yield per unit area, area, and yield of barley in Canada (unit: %).

	Disaster Year	Impact on Yield per Unit Area	Average Impact	Impact on Planting Acreage	Average Impact	Impact on Yield	Average Impact
Drought	1961	−35.07	−16.96	4.58	−3.52	−32.09	−20.53
	1984	−8.96		0.34		−8.87	
	1988	−6.84		−14.66		−20.62	
Flood	1974	−13.24	−7.94	2.67	−9.01	−10.91	−16.24
	1988	−6.84		−14.66		−20.62	
	1995	−1.11		−2.83		−4.36	
	1997	−6.53		2.66		−4.25	
	2002	−16.93		−21.80		−35.10	
	2006	−0.43		−15.15		−15.24	
	2014	−10.47		−13.99		−23.22	

**Table 7:** Simulation results of changes in China’s barley import trade (Scenario 1).

Source of Import	Basic Scenario		Simulated Scenario	
	Import Quantity (10^4^ Tons)	Import Prices (USD/Ton)	Import Quantity (10^4^ Tons)	Import Prices (USD/Ton)
Australia	157.35	232.83	91.76	429.61
Canada	227.13	204.49	236.93	206.90
France	188.08	193.37	155.55	196.14
Other countries	272.07	228.37	278.88	232.71

**Table 8:** Simulation results of changes in barley production and consumption in China (Scenario 1).

Scenario	Yield (10^4^ Tons)	Import Quantity (10^4^ Tons)	Consumption (10^4^ Tons)	Domestic Price (USD/Ton)
Basic scenario	90.12	844.62	934.74	1.99
Simulated scenario	91.24	763.11	854.35	2.07

**Table 9:** Simulation results of changes in China’s barley import trade (Scenario 2).

Source of Import	Basic Scenario		Simulated Scenario	
	Import Quantity (10^4^ Tons)	Import Prices (USD/Ton)	Import Quantity (10^4^ Tons)	Import Prices (USD/Ton)
Australia	157.35	232.83	91.79	429.97
Canada	227.13	204.49	204.30	207.00
France	188.08	193.37	192.13	196.25
Other countries	272.07	228.37	279.14	232.87

**Table 10:** Simulation results of changes in barley production and consumption in China (Scenario 2).

Scenario	Yield (10^4^ Tons)	Import Quantity (10^4^ Tons)	Consumption (10^4^ Tons)	Domestic Price (USD/Ton)
Basic scenario	90.12	844.62	934.74	1.99
Simulated scenario	91.28	767.37	858.64	2.07

## Data Availability

Data is contained within the article or Supplementary Material.

## Supplementary Materials

The following supporting information can be downloaded at: https://www.mdpi.com/article/10.3390/foods11111570/s1, Table S1: Barley harvested area and price elasticity of yield per unit area; Table S2: Parameter estimation results of barley inverse demand function equation; Table S3: Estimation results of parameters of China’s barley import equation.
