# Supplementary material for: Impact of Extreme Weather Disasters on China’s Barley Industry under the Background of Trade Friction—Based on the Partial Equilibrium Model
Source: Foods. 2022 May 26;11(11):1570. doi: 10.3390/foods11111570 (PMC9180676; doi:10.3390/foods11111570)
Supplement: Supplementary file 1 [file foods-11-01570-s001.zip › foods-1738414-supplementary.pdf]

Table S1. Barley harvested area and price elasticity of yield per unit area.

	Barley	Rice	Wheat	Corn	Potato	Soybean	Rapeseed
Elasticity of barley harvested area	0.1500	0.0000	0.0000	0.0000	0.0000	-0.0100	-0.0200
Elasticity of barley yield price	0.2000	-	-	-	-	-	-

Continued Table 1

	Peanut	Cotton	Sugarcane	Beet	Apple	Other fruits	Vegetables
Elasticity of barley harvested area	-0.0200	-0.0200	0.0000	-0.0100	-0.0100	-0.0100	-0.0500
Elasticity of barley yield price	-	-	-	-	-	-	-

Table S2. Parameter estimation results of barley inverse demand function equation.

	α^{DBP}	β^{DBP}_{PGDP}	β^{DBP}_{DDCN}	R^2
Parameter	1.2340<br>（1.2041）	0.3486***<br>（0.0588）	-0.3896*<br>（0.1965）	0.63

Table S3. Estimation results of parameters of China's barley import equation.

Import equation between China and Australia							
	α^{IM_a}	Q_{at}	DBP_t	IBP_{at}	DZ_{at}	DJ_{at}	R^2
Parameter	4.7064<br>(3.5696)	0.7175**<br>(0.3000)	0.8840**<br>(0.3943)	-0.9313*<br>(0.4793)	0.5327*<br>(0.2795)	0.1058<br>(0.3050)	0.54
**Import equation between China and Canada**							
	α^{IM_c}	Q_{ct}	DBP_t	IBP_{ct}	DZ_{ct}	DJ_{ct}	R^2
Parameter	12.7936***<br>(3.8871)	0.4826<br>(0.4826)	1.8508***<br>(0.4453)	-1.9765***<br>(0.5078)	0.5456**<br>(0.2479)	-0.6728**<br>(0.2561)	0.59
**Import equation between China and France**							
	α^{IM_f}	Q_{ft}	DBP_t	IBP_{ft}	DZ_{ft}	DJ_{ft}	R^2
Parameter	-3.5901<br>(10.2402)	2.5412*<br>(1.2983)	1.4127*<br>(0.6959)	-2.0637***<br>(0.6391)	0.5703<br>(0.4903)	-1.0182***<br>(0.3477)	0.70
**Import equation between China and other countries**							
	α^{IM_o}	Q_{ot}	DBP_t	IBP_{ot}	DZ_{ot}	DJ_{ot}	R^2
Parameter	7.6144<br>(23.3393)	0.6025<br>(2.3407)	1.7555*<br>(0.9030)	-1.9835*<br>(1.0091)	0.6858<br>(0.5929)	-0.1346<br>(0.5929)	0.32

Transfer equation of import price between China and Australia					
	α^{IBP_a}	DBP_t	EX_{at}	DCP_t	R^2
Parameter	4.0080***<br>(0.5081)	0.6229***<br>(0.1977)	0.6927**<br>(0.3037)	-0.0511<br>(0.2048)	0.81
**Transfer equation of import price between China and Canada**					

	α^{IBP_c}	DBP_t	EX_{ct}	DCP_t	R^2
Parameter	3.1915***<br>(0.5264)	0.3320*<br>(0.1727)	1.1233<br>(0.2932)	0.4610**<br>(0.1882)	0.81
**Transfer equation of import price between China and France**					
	α^{IBP_f}	DBP_t	EX_{ft}	DCP_t	R^2
Parameter	2.2037*<br>(1.0018)	0.4021<br>(0.2815)	1.3421***<br>(0.4592)	0.3458<br>(0.3084)	0.67
**Transfer equation of import price between China and other countries**					
	α^{IBP_o}	DBP_t	EX_{ot}	DCP_t	R^2
Parameter	6.7757***<br>(1.7543)	0.5327**<br>(0.2043)	-0.7649<br>(0.8246)	-0.044<br>(0.3066)	0.69

Note: Numbers in parentheses are t-statistics values.***-1% significance level;**-5%significance level;*-10% significance level
